# Extra-helical allosteric binding site of apomorphine in ADGRG6

**DOI:** 10.1038/s41421-025-00866-1

**Published:** 2026-02-03

**Authors:** Na Qiu, Wei Xu, Tuo Xu, Wenbin Xie, Youqi Jiang, Zhiwei Zhong, Limin Ma, Qiang Zhao, Beili Wu, Shuo Han

**Affiliations:** 1 State Key Laboratory of Drug Research, State Key Laboratory of Chemical Biology, Shanghai Institute of Materia Medica, Chinese Academy of Sciences, Shanghai, China; 2 School of Life Science and Technology, ShanghaiTech University, Shanghai, China; 3 Lingang Laboratory, Shanghai, China; 4 University of Chinese Academy of Sciences, Beijing, China; 5 School of Chinese Materia Medica, Nanjing University of Chinese Medicine, Nanjing, Jiangsu China; 6 Zhongshan Institute for Drug Discovery, Shanghai Institute of Materia Medica, Chinese Academy of Sciences, Zhongshan, Guangdong China; 7 School of Pharmaceutical Science and Technology, Hangzhou Institute for Advanced Study, University of Chinese Academy of Sciences, Hangzhou, Zhejiang China; 8 Shanghai Academy of Natural Sciences (SANS), Shanghai Institute of Materia Medica, Chinese Academy of Sciences, Shanghai, China

**Keywords:** Cryoelectron microscopy, Molecular biology

Dear Editor,

Adhesion G protein-coupled receptors (ADGRs), the second largest G protein-coupled receptor (GPCR) subfamily, orchestrate diverse physiological processes, including development, immune regulation, and nervous system function^1,2^. Within this family, ADGRG6 (also termed GPR126) serves as a central regulator of Schwann cell development and peripheral nervous myelination^3^. Dysregulation of ADGRG6 has been implicated in pathological conditions such as scoliosis^4,5^, congenital contractures^6^, and breast cancer^7^. Despite the growing physiological and pharmacological relevance of ADGRG6, the structural mechanism underlying ADGRG6 activation, particularly in response to small-molecule ligands, remains elusive. Pharmacological studies have identified apomorphine hydrochloride, a classical dopamine receptor agonist, as a modulator of ADGRG6 that promotes Schwann cell differentiation and myelination through G_s_-mediated signaling^8^. However, the molecular basis of apomorphine recognition and receptor activation has not been resolved.

To address this gap, we determined the cryo-electron microscopy (cryo-EM) structure of apomorphine-bound ADGRG6 in complex with miniGα_s_. The structure uncovers two distinct modes of receptor activation: one mediated by the tethered N-terminal stalk peptide, and another by apomorphine occupying a previously uncharacterized extra-helical allosteric pocket. Supported by mutagenesis and functional assays, these findings provide new insights into ADGRG6 activation and establish a structural framework for rational drug discovery targeting this receptor subfamily.

A truncated human ADGRG6 construct encompassing residues S578–L1136, which preserves the native autoproteolytic site and includes the GPCR autoproteolysis-inducing (GAIN) domain and the seven-transmembrane (7TM) core, was engineered to enhance protein stability and yield, and co-expressed with miniGα_s_ and Gβγ in High Five insect cells. Complex assembly was facilitated by adding apomorphine and the nanobody Nb35 during purification. Cryo-EM single-particle analysis yielded a reconstruction map at 2.9 Å resolution based on the gold-standard Fourier Shell Correlation (FSC) = 0.143 criterion (Fig. 1; Supplementary Figs. S1, S2 and Table S1), which enabled modeling of most residues of the 7TM domain (residues D857–Q1127), stalk peptide (residues T841–L856), G protein subunits (S13–L394 of miniGα_s_, S2–N340 of Gβ, and S8–R62 of Gγ), and the small-molecule ligand apomorphine. Although the full GAIN domain was included in the construct, it was not visualized, most likely due to its high conformational flexibility relative to the 7TM core, a phenomenon consistently observed in previously reported ADGR structures.

**Fig. 1:**
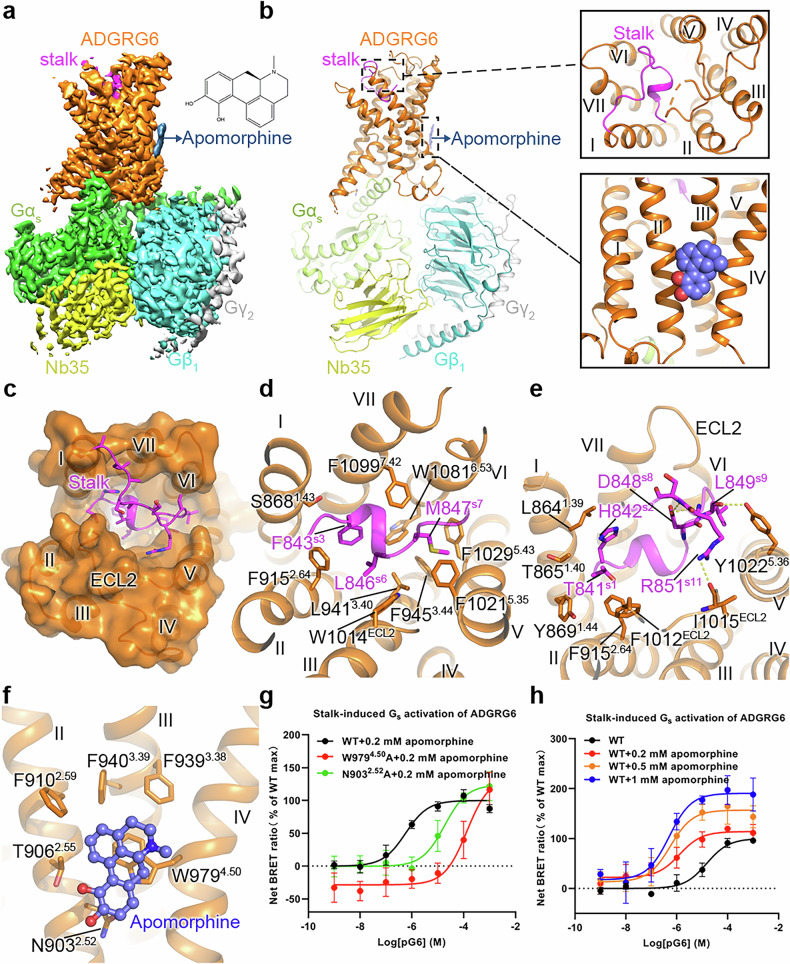
Cryo-EM structure of the apomorphine-bound ADGRG6–G_s_ complex, the mutagenesis study, and the extra-helical binding site of apomorphine. **a**, **b** Cryo-EM map and model of the apomorphine–ADGRG6–G_s_ complex. The stalk and transmembrane domain (TMD) of ADGRG6, Gα_s_, Gβ, Gγ, Nb35, and apomorphine are colored magenta, orange, limon, cyan, grey, yellow and blue, respectively. The binding cavities for the stalk peptide and apomorphine are highlighted by two dashed boxes and are shown in detail on the right (**b**). **c**–**e** Interaction mechanism between the stalk peptide and the TMD. **c** Stalk-binding cavity in ADGRG6. The receptor TMD in the ADGRG6–miniG_s_ structure is shown in orange in both cartoon and surface representations. The stalk is shown in magenta in a cartoon representation. **d** Interactions between the TMD and the stalk residues F^s3^, L^s6^ and M^s7^ in ADGRG6. The residues involved in the interactions are shown as sticks. **e** Interactions between the TMD and the stalk residues T841^s1^, H842^s2^, D848^s8^, L849^s9^ and R851^s11^ in ADGRG6. The residues involved in the interactions are shown as sticks. Polar interactions are shown as yellow dashed lines. **f** The extra-helical binding site of apomorphine in active ADGRG6. The ADGRG6–miniG_s_ structure is shown in cartoon representation. The small-molecule apomorphine is shown as blue sticks. The receptor residues involved in apomorphine binding are shown as orange sticks. **g**, **h** Stalk peptide (pG6)-induced G_s_ activation of ADGRG6. Data are shown as means ± SEM from at least three independent experiments performed in technical duplicate. Supplementary Table S2 provides detailed information on the number of independent experiments (*n*), statistical analyses, and expression levels.

The structure reveals that the stalk peptide occupies the orthosteric pocket within the transmembrane helical bundle (Fig. 1c). A short α-helix of the stalk peptide is stabilized through hydrophobic residues F843^s3^, L846^s6^ and M847^s7^ (superscripts indicate residue positions in the stalk, abbreviated as “s”), which penetrate deeply into a conserved ADGR pocket, forming interactions with F915^2.64^, L941^3.40^, F945^3.44^, W1014^ECL2^, F1021^5.35^, F1029^5.43^, W1081^6.53^ and F1099^7.42^ (superscripts refer to the Wootten numbering system for class B GPCRs) (Fig. 1d). These interactions resemble those observed in the active structures of ADGRG1, ADGRG2 and ADGRG4^9,10^, consistent with a conserved tethered peptide-mediated activation mechanism of the ADGRs (Supplementary Fig. S3a–c). Of note, additional contacts stabilize the stalk conformation at the upper region of the binding pocket, where residues T841^s1^ and H842^s2^ form hydrophobic interactions with the extracellular tips of helices I and II, and extracellular loop 2 (ECL2), engaging with residues L864^1.39^, T865^1.40^, Y869^1.44^, F915^2.64^, and F1012^ECL2^. Polar contacts further reinforce this arrangement, with L849^s9^ and R851^s11^ forming hydrogen bonds with Y1022^5.36^ and I1015^ECL2^, while D848^s8^ bridges the N- and C-terminal regions of the stalk peptide via a network of hydrogen bond and hydrophobic interactions, thereby stabilizing the stalk–receptor complex (Fig. 1e). Collectively, these coordinated interactions define the orientation of the tethered peptide and stabilize the active conformation of the stalk sequence.

Comparison with the inactive ADGRL3 structure (PDB: 8JMT) reveals that the stalk peptide induces substantial conformational rearrangements that propagate from the extracellular side to the intracellular G protein-binding interface (Supplementary Fig. S4a, b). At the base of the orthosteric binding pocket, insertion of F843^s3^ disrupts the hydrophobic triad formed by F915^2.64^, W1081^6.53^ and F1099^7.42^, directly displacing the “toggle switch” residue W1081^6.53^, a hallmark microswitch in GPCR activation (Supplementary Fig. S4c). This triggers an outward displacement of helix VI, especially around the F^6.47^XXG^6.50^ motif, accompanied by reorganization of interactions between W1081^6.53^ and residues F945^3.44^, M948^3.47^, F1029^5.43^, F1099^7.42^, N1103^7.46^ and Q1106^7.49^ (Supplementary Fig. S4d). These interaction rearrangements result in a clockwise outward rotation of helices VI and VII, as viewed from the extracellular side. In addition, insertion of the stalk peptide induces a displacement of helix I (Supplementary Fig. S4a, b).

Further comparison of active ADGRG6 with all previously reported active structures of ADGRs reveals a conserved activation mechanism: a pronounced kink in helices VI and VII (Supplementary Fig. S3d–f). In our previous studies of ADGRF1 and ADGRD1, we showed that this sharp bend in helix VI was mediated by the conserved P^6.47^XXG^6.50^ motif, consistent with active conformations observed in class B1 receptors. Even in other members such as ADGRL3, ADGRG1, ADGRG2, ADGRG4 and ADGRE5, where the proline residue is replaced by cysteine, leucine, or phenylalanine, unwinding and bending at the midpoint of helix VI remain conserved in all the active ADGR structures. Notably, glycine at this motif remains conserved across all members, underscoring its essential role in helix bending. Likewise, helix VII undergoes a characteristic kink at the Q^7.49^G^7.50^ motif upon stalk peptide insertion, although in ADGRG5 glutamine is substituted by tyrosine. These structures demonstrate that ADGRs adopt highly conserved conformational rearrangements upon stalk peptide activation, highlighting a general interaction pattern that facilitates G protein coupling.

The canonical class B GPCR motif P^6.47^XXG^6.50^ is substituted by F^6.47^LLG^6.50^ in ADGRG6, which creates an additional hydrophobic core involving F1075^6.47^, F1102^7.45^, and F1109^7.52^. The backbone of F^6.47^ forms a hydrogen bond with Q1106^7.49^, tightly linking the bends of helices VI and VII, while L^6.48^ and L^6.49^ engage with residues in helices III, V, and VII, including E951^3.50^, M955^3.54^, N1036^5.50^, F1040^5.54^, and H1114^7.57^, thereby further stabilizing the helical packing (Supplementary Fig. S4e). At the cytoplasmic side, the canonical E/DR^3.50^Y motif of class A GPCRs is replaced by an HM^3.54^Y motif in ADGRG6. The bulky side chain of H^3.53^ interacts with the residues Y896^2.45^, P897^2.46^, Y970^4.41^ and F974^4.45^ in helices II and IV, while simultaneously contacting the α5 helix of Gα_s_. Additionally, the side chain of M^3.54^ engages in hydrophobic interactions with M1039^5.53^ and L1077^6.49^ in helices V and VI (Supplementary Fig. S4f). Together, these interactions within transmembrane helices stabilize the open intracellular cavity, promoting efficient G protein engagement and signal transduction.

In addition to the tethered peptide-mediated activation mechanism, our structure reveals a previously uncharacterized extra-helical allosteric pocket that accommodates apomorphine (Fig. 1f). The ligand resides in a shallow cavity formed by helices II–IV, where its rigid four-ring scaffold defines the binding orientation, with the catechol moiety orienting towards the intracellular side and the tetrahydropyridine ring extending towards the extracellular side. The most prominent interaction is a face-to-face π–π stacking between apomorphine and residue W979^4.50^, which contributes to tight packing against helix IV (Fig. 1f). This interaction is further strengthened by hydrophobic contacts involving F910^2.59^, F939^3.38^ and F940^3.39^, which constitute the architecture of the pocket. Moreover, the hydroxyl groups of the catechol moiety engage in weak polar contacts with N903^2.52^ and T906^2.55^, providing complementary stabilization. Collectively, these coordinated hydrophobic and polar interactions define the binding pose of apomorphine within the extra-helical cavity and suggest a structural basis for allosteric modulation of ADGRG6 by small molecules. Although the apomorphine density is moderate — consistent with its relatively low affinity — it nonetheless reveals a characteristic feature at this extra-helical site, providing meaningful insights into ligand recognition.

To further elucidate the mechanism of apomorphine recognition and activation of ADGRG6, site-directed mutagenesis was performed for some key residues, followed by functional assessment using a bioluminescence resonance energy transfer (BRET) assay (Fig. 1g; Supplementary Table S2). The results showed that alanine substitution of W979^4.50^, which abolishes the critical π–π stacking interaction, resulted in an ~234-fold reduction of the agonistic potency of the synthetic stalk peptide (pG6: T^841^HFGVLMDLPRSASQL^856^) in inducing G_s_ activation in the presence of 0.2 mM apomorphine. Similarly, replacement of N903^2.52^ to alanine decreased the potency by 32-fold, underscoring the functional importance of this polar contact for receptor activation. These results identify W979^4.50^ and N903^2.52^ as principal determinants for apomorphine engagement and signaling. Furthermore, to assess whether these mutations affect orthosteric activation, we examined stalk peptide-mediated G_s_ activation for W979^4.50^A and N903^2.52^A mutants in the absence of apomorphine. Both mutants exhibited comparable EC_50_ and *E*_max_ values to the wild-type (WT) receptor (Supplementary Fig. S4h, i and Table S2), indicating that these residues are not required for stalk peptide-driven activation. Together with the observed loss of apomorphine-enhanced signaling in these mutants, these results demonstrate that W979^4.50^ and N903^2.52^ specifically contribute to the allosteric modulation by apomorphine rather than its orthosteric signaling. In addition, we mutated residues within the orthosteric pocket and evaluated their effects on apomorphine-induced G_s_ protein activation. Of note, F945^3.44^A, W1014^ECL2^A, Y1022^5.36^A, and F1029^5.43^A mutants displayed signaling responses comparable to those of the WT receptor (Supplementary Fig. S4g and Table S2). These results indicate that canonical orthosteric pocket residues contribute minimally to apomorphine-mediated activation, thereby further supporting the extra-helical allosteric binding mode for apomorphine in ADGRG6.

To further evaluate the functional consequences of apomorphine binding, we conducted BRET-based G_s_ protein activation assays. Apomorphine enhanced the stalk peptide-induced activation of G_s_ protein (Fig. 1h; Supplementary Table S2), resulting in both enhanced agonistic potency and an elevated maximal signaling response. These findings indicate that apomorphine functions as a positive allosteric modulator (PAM), exerting functional cooperativity with the endogenous stalk peptide. The engagement of apomorphine appears to stabilize the active conformation of ADGRG6. Positioned adjacent to transmembrane helices II, III, and IV, apomorphine engages a network of surrounding residues, including N903^2.52^, F906^2.55^, F910^2.59^, F939^3.38^, F940^3.39^, and W979^4.50^, which together constitute the extra-helical cavity. Through these contacts, apomorphine likely promotes tighter packing of the helices II–IV interface and attenuates local conformational flexibility. This stabilization is expected to propagate across the helical bundle, thereby restricting helical mobility and consolidating the conformational rearrangements initiated by the stalk peptide, which collectively define the active architecture of the receptor. Quantitative fitting with an operational allosteric model indicated a detectable, low-affinity interaction that nonetheless supported a cooperative mechanism of receptor activation. These findings demonstrate that ADGRG6 can be modulated by small molecules through a structurally defined extra-helical pocket, thereby providing a mechanistic framework for the rational design of more potent modulators.

Sequence alignment revealed that the key residues involved in apomorphine recognition are highly conserved across the ADGRG subfamily, particularly N^2.52^ and W^4.50^, suggesting that the extra-helical binding pocket defined in the ADGRG6 structure may represent a conserved allosteric ligand-binding pocket (Supplementary Fig. S5a). To further verify whether this allosteric mechanism is conserved across the ADGRG subfamily, we examined the effect of apomorphine on other family members, including ADGRG1, ADGRG2, and ADGRG4. In all three receptors, apomorphine enhanced stalk peptide-induced signaling, resulting in increased maximal responses (Supplementary Fig. S5b–d). These findings demonstrate that apomorphine acts as a PAM not only for ADGRG6 but also for other ADGRG receptors, suggesting a conserved allosteric regulation mechanism within this subfamily. Given that the critical residues forming the extra-helical binding pocket are conserved among ADGRG receptors, these findings collectively define a structurally conserved pocket capable of accommodating small-molecule modulators and suggest a shared mechanism of allosteric modulation across the ADGRG subfamily. Additionally, through in vivo and in vitro screening assays, the apomorphine alkaloid glaucine was identified to interact with ADGRG6^8^. To elucidate its binding mode, molecular docking was performed. The results suggest that, similar to apomorphine, glaucine also occupies the extra-helical allosteric binding site, and establishes extensive contacts with surrounding residues (Supplementary Fig. S6). In particular, the four-ring scaffold of glaucine engages in a prominent face-to-face π–π stacking interaction with W979^4.50^, while one of its methoxy group forms hydrophobic interactions with F910^2.59^ and F940^3.39^. These interactions anchor glaucine within the extra-helical pocket and stabilize the complex active conformation. Together, these findings imply the extra-helical cavity as a structurally conserved and pharmacologically accessible allosteric site, thereby providing a framework for the rational design of new modulators with improved potency and selectivity for the ADGRs.

In conclusion, we report the cryo-EM structure of the apomorphine–ADGRG6–miniG_s_ complex and elucidate the molecular mechanisms of receptor activation by both tethered and small-molecule agonists. We acknowledge that the low affinity of apomorphine and the moderate ligand density observed in the cryo-EM map introduce certain limitations to ligand assignment. Nevertheless, the combined structural features and supporting functional data consistently indicate the existence of an extra-helical allosteric site capable of accommodating small-molecule modulators. Previous crystal structures of the ADGRG6 ectodomain (PDB: 6V55) have shown that the GAIN domain mediates autoproteolysis and shields the tethered stalk peptide, maintaining the receptor in an autoinhibited state. Our cryo-EM structure of the 7TM–miniG_s_ complex complements these findings by revealing how the exposed stalk engages the orthosteric pocket to induce intracellular conformational rearrangements. Integrating these structural insights suggests that ligand binding or mechanical perturbation disrupts the autoinhibited ectodomain, allowing the stalk peptide to insert into the 7TM core and activate G protein signaling. This activation process may be further modulated by transmembrane allosteric ligands such as apomorphine, which can lower the energy barrier for stalk peptide-mediated activation and enhance G protein coupling efficiency. The identification of the extra-helical allosteric binding site in ADGRG6 not only sheds light on the binding mode of small-molecule allosteric ligands but also offers a new pharmacological avenue for targeting ADGRG6 in demyelinating diseases and beyond.

## Supplementary information

Supplementary Information

## Data Availability

The atomic coordinates and the electron microscopy maps of the apomorphine-bound ADGRG6–Gs complex have been deposited in the Protein Data Bank (PDB) under the accession code 21DU and Electron Microscopy Data Bank (EMDB) under the accession code EMD-67602, respectively.
