# Supplementary material for: Extra-helical allosteric binding site of apomorphine in ADGRG6
Source: Cell Discov. 2026 Feb 3;12:9. doi: 10.1038/s41421-025-00866-1 (PMC12868696; doi:10.1038/s41421-025-00866-1)
Supplement: Supplementary file 1 — Supplementary Information [file 41421_2025_866_MOESM1_ESM.pdf]

Materials and Methods

Construct cloning

The codon-optimized gene of human ADGRG6 (residues S578-L1136) was cloned into a modified pFastBac1 vector (Invitrogen) with haemagglutinin (HA) signal peptide at the N terminus. To facilitate expression and purification, the Flag and strep tags were added to the C termini of ADGRG6. The dominant-negative miniGα_s was engineered by introducing multiple mutations (G49D, E50N, A249D, S252D, L272D, I372A, and V375I) to enhance the stability of the heterotrimeric G protein complex. In addition, the N-terminal region of miniGα_s (residues L4–K25) was replaced with the corresponding N-terminal residues of miniGα_{i1} (residues T4–M18). The modified miniGα_s gene was cloned into the pFastBac1 vector, while human Gβ_1 (with an N-terminal hexa-histidine tag) and Gγ_2 were subcloned into the pFastBac Dual vector (Invitrogen).

Expression and purification of ADGRG6–miniG$_s$ protein complex

The miniG$_s$ protein-bound ADGRG6 complex was obtained by co-expressing the receptor, miniGα_s and G$\beta_1\gamma_2$ in High Five insect cells (Invitrogen). High-titer recombinant viral stocks were generated using the Bac-to-Bac Baculovirus Expression System (Invitrogen) and were used to transfect insect cells at a multiplicity of infection (MOI) ratio of 2:3:3. The transfection was performed at a cell density of 1.5×10^6 cells/mL, and the cells were cultured at 27 °C for 48 hours before harvesting.

The cells expressing the ADGRG6–miniG$_s$ protein complex were collected by

centrifugation and resuspended in a buffer containing 20 mM HEPES, pH 7.5, 50 mM NaCl, 2 mM $MgCl_2$, and EDTA-free protease inhibitor cocktail tablets (Roche) using dounce homogenization. The membrane suspension was supplemented with 25 mU/mL apyrase and incubated at room temperature for 2 h. Membrane pellets were then collected by centrifugation at 20,000g for 30 min. The protein complex was extracted from the membranes by incubating them with a solubilization buffer containing 20 mM HEPES, pH 7.5, 150 mM NaCl, 2 mM $MgCl_2$, 0.5% (w/v) lauryl maltose neopentyl glycol (LMNG, Anatrace), and 0.05% (w/v) cholesteryl hemisuccinate (CHS, Anatrace) at 4 °C for 2 h. The solubilized complex was separated by centrifugation at 30,000g for 30 min, and the supernatant was incubated overnight at 4 °C with Strep-Tactin XT Sepharose resin (IBA Lifesciences) and small molecule ligand apomorphine hydrochloride (200 μm, Sigma). To enhance complex stability, a 1.5-fold molar excess of Nb35 (protocols for its expression and purification are provided below) was added during the membrane solubilization process.

The resin was collected by centrifugation at 800g for 5 min and washed with 4 column volumes of 20 mM HEPES, pH 7.5, 150 mM NaCl, 2 mM $MgCl_2$, 200 μm apomorphine, 0.01% (w/v) LMNG and 0.001% (w/v) CHS to decrease the LMNG concentration. Detergent exchange was performed by incubating with 20 mM HEPES, pH 7.5, 150 mM NaCl, 2 mM $MgCl_2$, 200 μm apomorphine and 0.25% (w/v) glyco-diosgenin (GDN, Anatrace) at 4 °C for 2 h. The resin was collected again and washed with 10 column volumes of 20 mM HEPES, pH 7.5, 150 mM NaCl, 2 mM $MgCl_2$, 200 μm apomorphine and 0.01% (w/v) GDN. The complex protein bound to the strep resin

was eluted with 5 column volumes of 200 mM Tris-HCl, pH 8.0, 150 mM NaCl, 2 mM $MgCl_2$, 200 μm apomorphine, 0.01% (w/v) GDN and 50 mM biotin. The eluted protein was further incubated with 200 μm apomorphine hydrochloride (Sigma) at 4 °C for 5 h, followed by incubation with Ni-NTA resin (Clontech) for 1 h. The resin was collected and washed with 10 column volumes of 20 mM HEPES, pH 7.5, 150 mM NaCl, 2 mM $MgCl_2$, 200 μm apomorphine, 0.01% (w/v) GDN and 30 mM imidazole. The complex protein was then eluted with the same buffer supplemented with 300 mM imidazole and loaded to size-exclusion chromatography (SEC) using a Superdex 200 Increase 10/300 GL column (GE Healthcare) pre-equilibrated in 200 μm apomorphine, 20 mM HEPES, pH 7.5, 150 mM NaCl, 2 mM $MgCl_2$ and 0.01% (w/v) GDN. The complex fractions were pooled and concentrated to 3 mg ml^{-1} with a 100-kDa molecular weight cut-off concentrator (Millipore) for cryo-EM experiments. The protein purity and homogeneity were analyzed using SDS-PAGE and analytical SEC.

Expression and purification of Nb35

Nanobody35 (Nb35) was expressed and purified as previously described with modifications. Briefly, the C-terminal 6×His-tagged Nb35 gene was cloned into pET28a vector and expressed in Escherichia coli stain BL21 (DE3). The cells were cultured in LB media supplemented with 50 μg ml^{-1} kanamycin at 37 °C until OD_{600} reaching 0.6. Then the cultures were induced by 1 mM IPTG, and grown at 16 °C for 12 h. The cell pellets were harvested by centrifugation at 4,000g for 30 min and then lysed in 10 mM HEPES, pH 7.5 and 100 mM NaCl by sonication. The supernatant was isolated by centrifugation at 30,000g for 30 min, and incubated with Ni-NTA resin at

4 °C for 1 h. The resin was then washed with 20 column volumes of 10 mM HEPES, pH 7.5, 100 mM NaCl and 30 mM imidazole. The Nb35 protein was eluted by 10 column volumes of 10 mM HEPES, pH 7.5, 100 mM NaCl and 300 mM imidazole, and then submitted to SEC using a Superdex 75 10/300 GL column (GE Healthcare) in 10 mM HEPES, pH 7.5 and 100 mM NaCl. Peak fractions were pooled together and concentrated to 3 mg ml^{-1}. The final Nb35 sample was supplemented with 10% glycerol and stored at –80 °C until use.

Cryo-EM data acquisition

The formation of ADGRG6–miniG_s protein complex was confirmed by negative staining EM and the sample quality was evaluated by a 200 kV Talos Arctica G2 electron microscope (FEI). To prepare the cryo-EM grids, 3 μl of purified complex sample was applied to freshly glow-discharged amorphous NiTi foil R1.2/1.3 300 mesh Au grids and followed by the vitrification via plunge-frozen in liquid ethane cooled by liquid nitrogen using a FEI Vitrobot Mark IV (ThermoFisher Scientific) with 1.5 s blot time and 0 blot force at 4 °C and 100% humidity. The well-prepared grids were selected for data acquisition by using a 300 kV Titan Krios G3 electron microscope (FEI) equipped with a K3 Summit direct electron detector (Gatan) at a nominal magnification of 81,000× and a GIF-Quantum LS Imaging energy filter with a slit width of 20 eV. Images were captured by SerialEM software with a physical pixel size of 1.071 Å and a defocus ranging from –0.8 μm to –1.5 μm. Each image stack comprised 40 frames in a total of 3 s with 0.075 s exposure per frame, and the total dose was 70 electrons per $Å^2$.

Cryo-EM data processing and map construction

The image stacks of the ADGRG6–miniG$_s$ protein complex were subjected to beam-induced motion correction by MotionCor2. The contrast transfer function parameters of each micrograph were estimated using CTFFIND4 in CryoSPARC. The following data processing procedures were also performed by CryoSPARC. The resolution of density map was calculated by the gold-standard Fourier shell correlation (FSC) with the 0.143 criterion. After sharpening by post-processing in RELION 4.0, ResMap v.1.1.4 was used to estimate the local resolution.

For ADGRG6–miniG$_s$ complex, a total of 5,498 movies were collected and subjected to beam-induced motion correction and CTF determination. 4,056,558 particle projections were produced by reference-free Auto-picking and subjected to two rounds of 2D classification to discard false-positive particles. After that, 1,962,796 particles were served as a template to pick particles from the entire dataset. In total, 6,093,690 particles were picked and extracted for further 2D classification, ab initio reconstruction, and heterogeneous refinement. After removing the class of blurry particles, the best-resolved class, containing 1,233,868 particles, was subjected to Hetero refinement using cryoSPARC. Subsequently, a subset of 570,579 particles underwent non-uniform refinement in cryoSPARC and Bayesian polishing in RELION 4.0. The final refinement was completed using cryoSPARC, which yields a final map with global resolution of 2.9 Å.

Model building and refinement

The model of the ADGRG6–miniG$_s$ protein complex was built by recruitment of the receptor from AlphaFold predicted model, the subunits of Gα_s, Gβ_1, Gγ_2 and Nb35 from the ADGRF1–miniG$_s$ structure (PDB:7WU3) as initial template. Each model was docked into the corresponding cryo-EM density map by Chimera, followed by iterative manual adjustment in COOT and real-space refinement in phenix.real_space_refine of PHENIX. The model statistics were validated using Molprobity. The refined model exhibits high stereochemical quality, with 97.7% of residues in favored Ramachandran regions and an overall MolProbity score of 1.48. Structural figures were prepared by ChimeraX or PyMOL (https://pymol.org/2/) . The final refinement statistics are provided in Supplementary Table S1.

BRET assay using TRUPATH biosensors

To investigate the stalk-peptide (pG1, pG2, pG4, pG6) and apomorphine-induced G protein activation of ADGRG1, ADGRG2, ADGRG3 and ADGRG6, a bioluminescence resonance energy transfer (BRET) assay using TRUPATH biosensors was performed. The sequence of the peptides are as follows: pG1 (T^{383}YFAVLMVS^{391}), pG2 (T^{607}SFGVLLDLSR^{617}), and pG4 (T^{2722}HFGVLMDLSRSTVD^{2736}). This assay measured the proximity between RLuc8 fused to the Gα subunit and GFP2 fused to the Gγ subunit. The TRUPATH biosensors (Gα-RLuc8, Gβ, and Gγ-GFP2) were obtained from Addgene (kit no. 1000000163) as a gift from Dr. B. Roth, following a previously described protocol. HEK293F cells were transiently co-transfected with plasmids encoding wild-type ADGRG1, ADGRG2, ADGRG3 and ADGRG6 or mutant ADGRG6, along with Gα-RLuc8, Gβ_3, and Gγ_9-GFP2, at a ratio of 2:1:1:1. The

transfection was conducted at a cell density of 1.2 × 10^6 cells/mL with a total plasmid amount of 2 μg. After 48 hours, cell surface expression of receptors was assessed using a PE-conjugated anti-DDDDK tag antibody (ABclonal, 1:200 dilution in TBS with 4% BSA) at 4 °C for 20 minutes. The fluorescent signal was measured using flow cytometry (Guava easyCyte HT, Millipore), with compensation applied to subtract the GFP signal. For the BRET assay, transfected cells were plated in 96-well white plates at a density of 160,000 cells per well in 60 μL of assay buffer (HBSS with 20 mM HEPES, pH 7.4) and incubated at 37 °C for 30 minutes. A total of 10 μL freshly prepared 50 μM coelenterazine 400a (Nanolight Technologies) was added, and after 10 minutes of equilibration, BRET baselines were recorded using a Synergy II plate reader (Bio-Tek) with emission filters of 410 nm (RLuc8-coelenterazine 400a) and 515 nm (GFP2) for 15 minutes. The cells were then stimulated with 30 μL of synthesized stalk peptide (GL Biochem) or apomorphine hydrochloride (Sigma) at varying concentrations, and BRET signals were continuously monitored for five cycles. The BRET ratios were calculated as the ratio of GFP2 emission to RLuc8 emission and the data were analyzed using GraphPad Prism 8.0.

Molecular docking

The structure of ADGRG6 was used as the molecular docking models. The structure of the receptor was processed by using the 'Protein Preparation Wizard'. The ligands were converted from 2D to 3D structures using 'LigPrep' (Schrödinger). And molecular docking was carried out using 'Induced Fit Docking'.

Supplementary Data

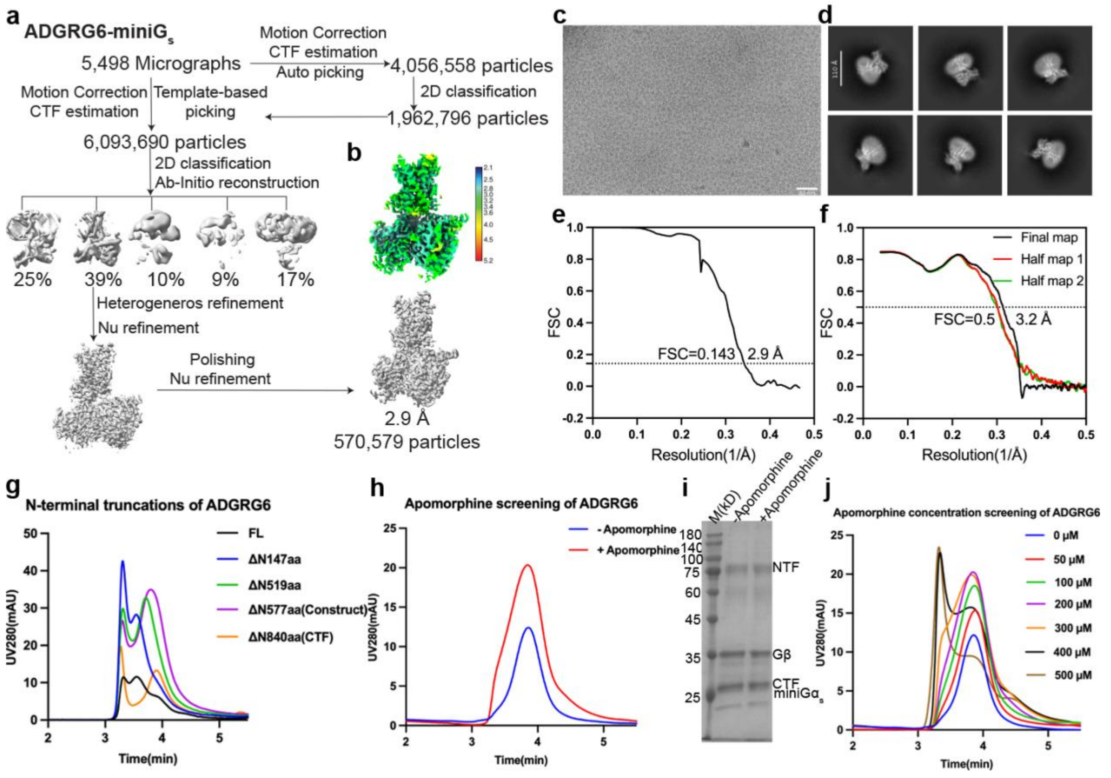

Supplementary Fig. S1. Cryo-EM processing, 3D reconstruction workflow and complex optimization of ADGRG6–miniG$_\text{s}$. a Data processing workflow. **b** Cryo-EM map colored according to local resolution (in Å). **c** Representative cryo-EM image. **d** Two-dimensional averages. **e** Gold-standard Fourier shell correlation (FSC) curve showing an overall resolution of 2.9 Å. **f** Cross-validation of model to cryo-EM density map. FSC curves for the final model versus the final map and half maps are shown in black, red and green, respectively. **g** SEC data of ADGRG6 N-terminal truncations screening of ADGRG6-G$_\text{s}$ complex. **h** SEC data of apomorphine screening of ADGRG6-G$_\text{s}$ complex. **i** SDS-PAGE result of apomorphine screening of ADGRG6-G$_\text{s}$ complex. **j** SEC data of apomorphine concentration screening of ADGRG6-G$_\text{s}$ complex.

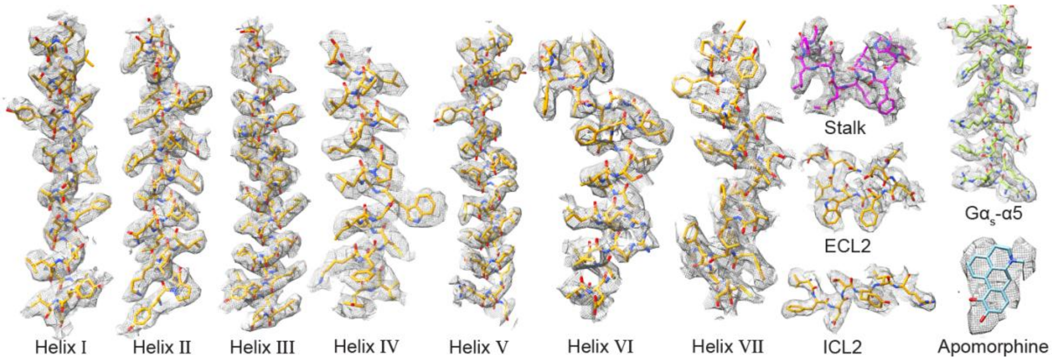

Supplementary Fig. S2. Cryo-EM density maps of the miniG$_s$-bound ADGRG6 structure. Cryo-EM maps and models of the miniG$_s$-bound ADGRG6 structure are shown for all the transmembrane helices, stalk, ECL2, ICL2, apomorphine and Gα$_s$ α5-helix. The models are shown as sticks. The maps are colored grey.

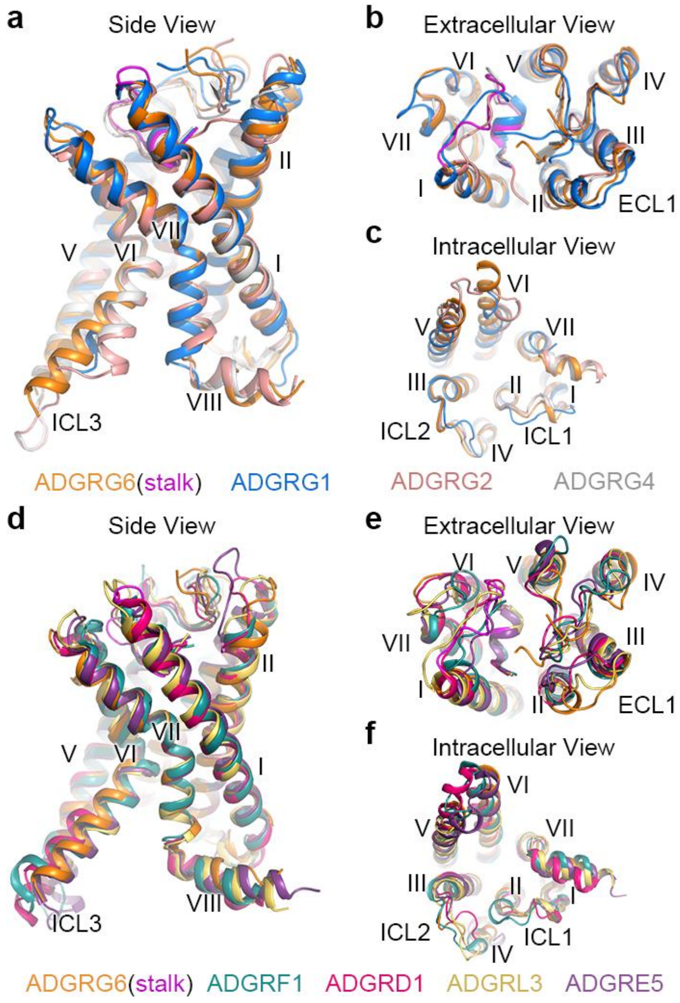

Supplementary Fig. S3. Comparison of the miniG$_s$-bound ADGRG6 structure with ADGRG1-G$_{13}$, ADGRG2/G4–G$_s$, ADGRF1-miniG$_s$, ADGRD1-miniG$_s$, ADGRL3–G$_s$ and ADGRE5–G$_s$ protein structures. a Structural comparison of the CTF in ADGRG1-G$_{13}$, ADGRG2/G4/G6–G$_s$ protein structures. The receptors in the structures of ADGRG1-G$_{13}$ (PDB: 7SF8), ADGRG2–G$_s$ (PDB: 7WUQ), ADGRG4–G$_s$ (PDB: 7WUJ) and ADGRG6–G$_s$ are shown in cartoon representation, and colored marine, pink, grey and orange, respectively. The stalks in the four receptors are colored marine, pink, grey and magenta, respectively. The G protein subunits are not displayed for clarity. **b, c** Structural comparison of the helical bundles of ADGRG1, ADGRG2, ADGRG4, and ADGRG6 from the extracellular **(b)** and intracellular **(c)** perspectives. **d** Structural comparison of the CTF in ADGRG6/F1/D1/L3/E5–G$_s$ protein structures. The receptors in the structures of ADGRF1-miniG$_s$ (PDB: 7WU3), ADGRD1-miniG$_s$ (PDB: 7WU2), ADGRL3–G$_s$

(PDB: 7WY8), ADGRE5–G_s (PDB: 7YDP) and ADGRG6–G_s are shown in cartoon representation, and forest, red, yellow, purple and orange, respectively. The G protein subunits are not displayed for clarity. **e,f** Structural comparison of the helical bundles of ADGRF1, ADGRD1, ADGRL3, ADGRE5, and ADGRG6 from the extracellular **(e)** and intracellular **(f)** perspectives.

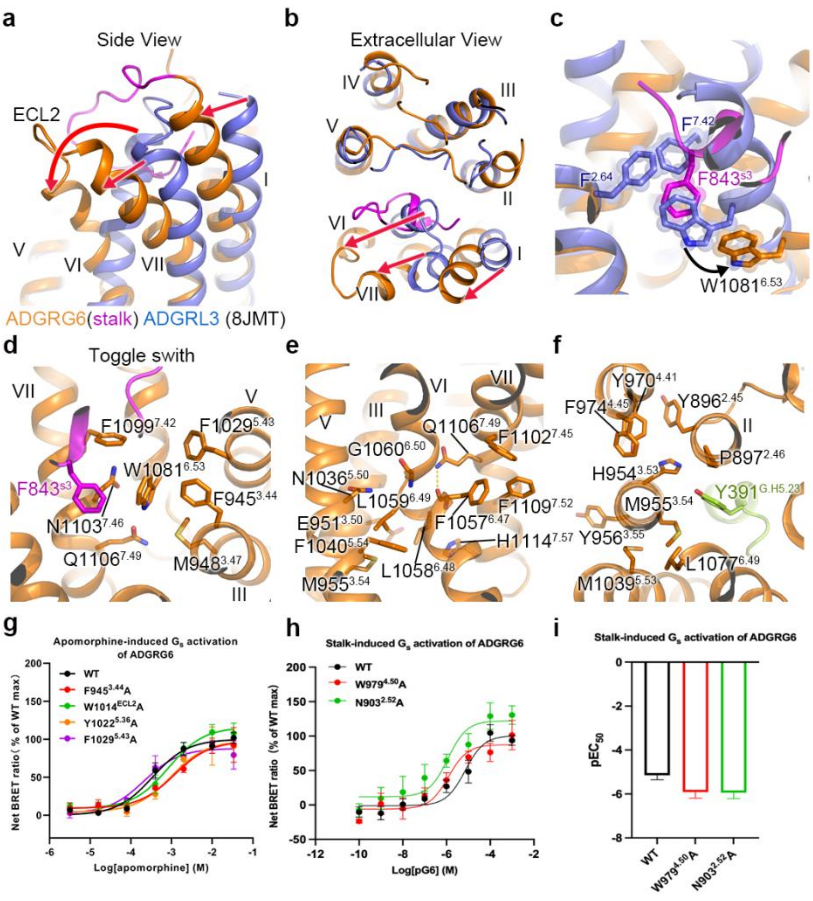

Supplementary Fig. S4. The activation conformation of ADGRG6. a The side view of ADGRG6–miniG$_s$ and inactive ADGRL3 (PDB: 8JMT), which was colored by orange and blue, respectively. The red arrows indicate the conformational differences of helices I, VI and VII in the G$_s$-bound active ADGRG6 relative to the inactive ADGRL3. **b** The extracellular view of ADGRG6-miniG$_s$ compared to inactive ADGRL3. The red arrows indicate the conformational differences of helices I, VI and VII in the G$_s$-bound active ADGRG6 relative to the inactive ADGRL3. **c** Compared with inactive ADGRL3, active ADGRG6 exhibits conformational changes in residues F$^{2.64}$, W$^{6.53}$, and F$^{7.42}$ induced by stalk peptide insertion. Residues involved in interactions are shown as sticks. **d-f** Interactions within toggle switch (**d**), F$^{6.47}$XXG$^{6.50}$ motif (**e**) and HM$^{3.54}$Y motif (**f**) in ADGRG6. Residues involved in the

interactions are shown as magenta sticks for the stalk, orange sticks for the receptor, and limon-colored sticks for the Gα_s protein. Polar interactions are shown as yellow dashed lines. **g** Apomorphine-induced G_s activation of ADGRG6. **h** Stalk peptide (pG6)-induced G_s protein activation of ADGRG6. **i** Potency of the peptide-induced G_s protein activation of ADGRG6. Data are shown as mean ± s.e.m. from at least three independent experiments performed in technical duplicate. Supplementary table S2 provides detailed independent experiment numbers (n), statistical evaluation and expression level.

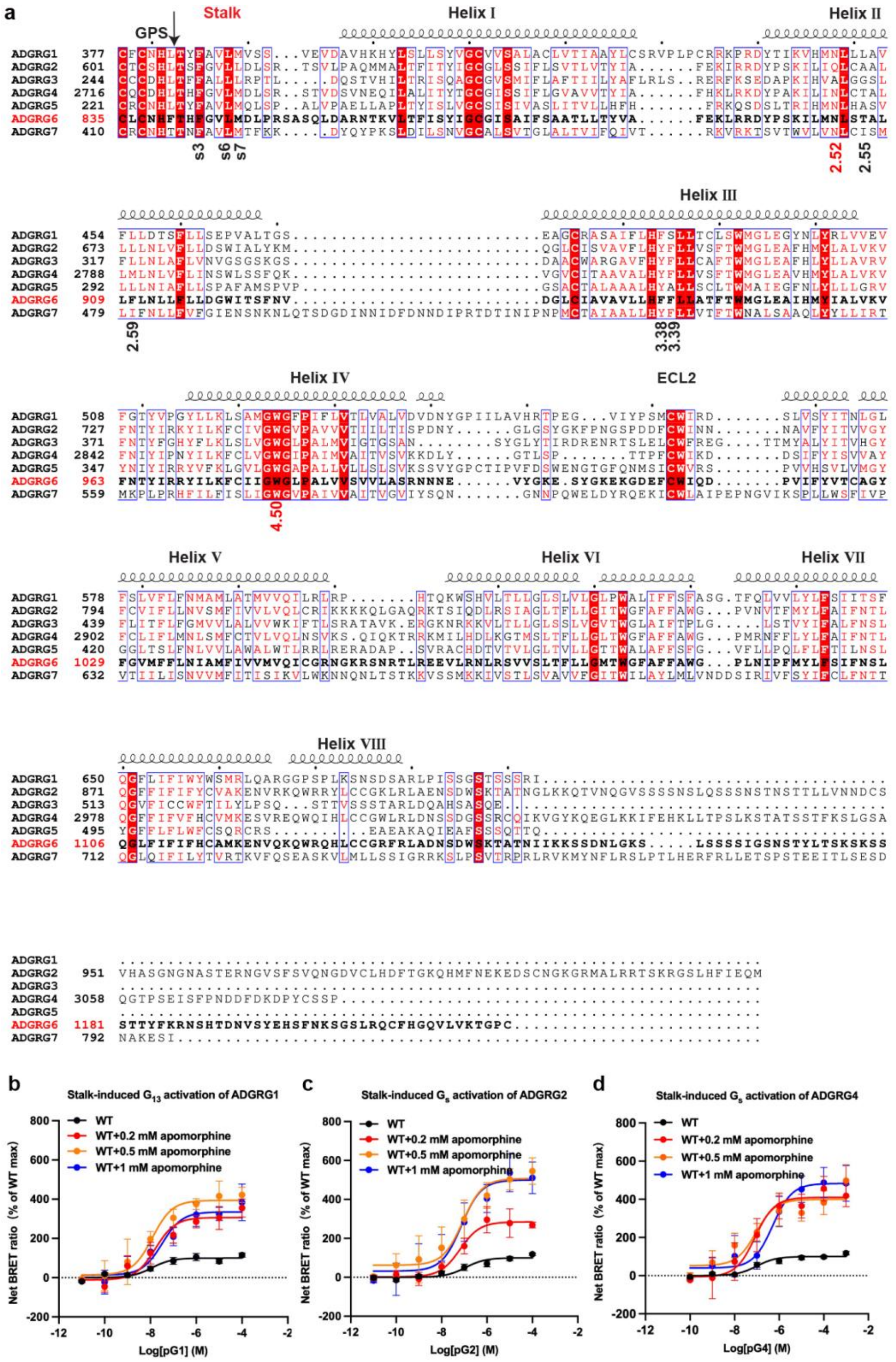

Supplementary Fig. S5. Multiple sequence alignment of adhesion GPCRs and stalk peptide-induced G protein activation of ADGRG1, ADGRG2 and ADGRG4. **a** Key positions in the stalk peptide and TMD are labeled below the sequence. The alignment was generated using using Clustal W program and the graphic was prepared on the ESPript 3.0 server (http://espript.ibcp.fr/ESPript/cgi-bin/ESPript.cgi). **b** stalk peptide (pG1)-induced G_{13} protein activation of ADGRG1. **c** stalk peptide(pG2)-induced G_s protein activation of ADGRG2. **d** stalk peptide

(pG4)-induced G_s protein activation of ADGRG4. Data are shown as mean ± s.e.m. from at least three independent experiments performed in technical duplicate. Supplementary table S2 provides detailed independent experiment numbers (n), statistical evaluation.

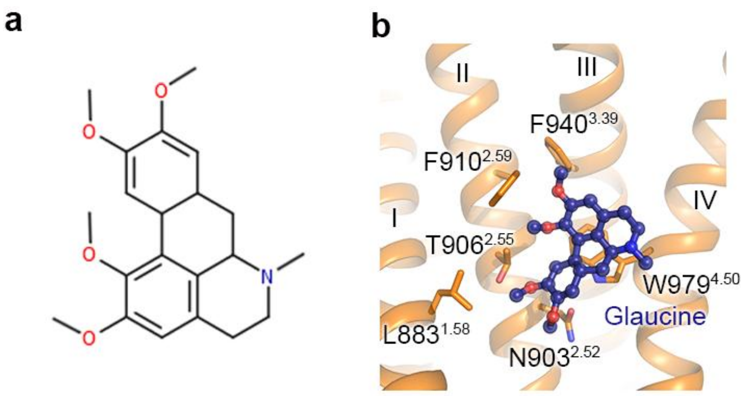

Supplementary Fig. S6. The 2D structure and docking pose of glaucine. a Chemical structure of glaucine. **b** Docking pose of glaucine in ADGRG6. The ADGRG6 structure is shown in cartoon representation. The small molecule apomorphine is shown as dark-blue sticks. The receptor residues involved in apomorphine binding are shown as orange sticks.

Supplementary Table S1. Cryo-EM data collection, refinement, and validation statistics

	**ADGRG6–miniG_s**
Magnification	81,000
Voltage (kV)	300
Electron exposure (e^-/Å^2)	70
Defocus range (µm)	–0.8 ~ –1.5
Pixel size (Å)	1.071
Symmetry imposed	C1
Initial particle images (no.)	4,056,558
Final particle images (no.)	570,579
Map resolution (Å)	2.9
FSC threshold	0.143
Map resolution range (Å)	2.0-5.0
Initial model used (PDB code)	7WU3
Model resolution (Å)	3.2
FSC threshold	0.5
Map sharpening *B* factor (Å^2)	-92.9
Model composition	
Non-hydrogen atoms	8,058
Protein residues	1039
B factors (Å^2)	
Protein	35.05
Ligand	32.91
R.m.s. deviations	
Bond lengths (Å)	0.004
Bond angles (°)	0.898
Validation	
Molprobity score	1.48
Clashscore	7.44
Poor rotamers (%)	0.36
Ramachandran plot	
Favored (%)	97.66
Allowed (%)	2.34
Disallowed (%)	0.00

Supplementary Table S2. G_s protein activation of wild-type ADGRG6 and mutants, measured by BRET assay

Apomorphine-induced G_s activation of ADGRG6

Mutants[†]	EC_{50} (μM)	EC_{50} ratio[‡]	pEC50		E_{max}[‖](% of WT)		n[¶]	Expression[#](% of WT)	
			mean ± s.e.m.[§]	*P* value	% of WT[§]	*P* value		% of WT[§]	*P* value
**WT**	340	1	3.47 ± 0.12	/	100 ± 6	/	10	100	/
**F945$^{3.44}$A**	1180	3	2.93 ± 0.20	0.0977	88 ± 9	0.7318	4	97 ± 4	0.9038
**W1014^{ECL2}A**	842	2	3.07 ± 0.15	0.2052	107 ± 9	0.9128	6	91 ± 1	0.1029
**Y1022$^{5.36}$A**	981	3	3.01 ± 0.21	0.1909	94 ± 10	0.9587	4	99 ± 6	0.9978
**F1029$^{5.43}$A**	211	1	3.67 ± 0.24	0.8662	86 ± 10	0.6677	3	102 ± 4	0.9745

Stalk peptide (pG6)-induced G_s activation of ADGRG6

ADGRG6	EC_{50} (μM)	EC_{50} ratio[‡]	pEC50		E_{max}[‖](% of WT)		n[¶]
			mean ± s.e.m.[§]	*P* value	% of WT[§]	*P* value	
**WT**	14.96	1	4.83 ± 0.22	/	100 ± 10	/	12
**WT(0.2 mM apomorphine)**	1.28	0.09	5.89 ± 0.34*	0.0336	92 ± 14	0.9740	5
**WT(0.5 mM apomorphine)**	0.57	0.04	6.25 ± 0.31*	0.0022	144 ± 20	0.1210	6
**WT(1 mM apomorphine)**	0.50	0.03	6.30 ± 0.29*	0.0029	173 ± 23*	0.0082	5

Stalk peptide (pG1)-induced G_{13} activation of ADGRG1

ADGRG1	EC_{50} (nM)	EC_{50} ratio[‡]	pEC50		E_{max}[‖](% of WT)		n[¶]
			mean ± s.e.m.[§]	*P* value	% of WT[§]	*P* value	
**WT**	11.45	1	7.94 ± 0.24	/	100 ± 10	/	8
**WT (0.2 mM apomorphine)**	16.34	1.43	7.79 ± 0.26	0.9605	318 ± 34***	<0.0001	4
**WT (0.5 mM apomorphine)**	12.18	1.06	7.91 ± 0.27	0.9998	380 ± 44***	<0.0001	4
**WT (1 mM apomorphine)**	32.44	2.83	7.49 ± 0.30	0.5225	322 ± 39***	<0.0001	4

Stalk peptide (pG2)-induced G_s activation of ADGRG2

ADGRG2	EC_{50} (nM)	EC_{50} ratio[‡]	pEC50		E_{max}[‖](% of WT)		n[¶]
			mean ± s.e.m.[§]	*P* value	% of WT[§]	*P* value	
**WT**	112.93	1	6.95 ± 0.22	/	100 ± 9	/	12
**WT (0.2 mM apomorphine)**	65.06	0.58	7.19 ± 0.27	0.9210	282± 31*	0.0234	4
**WT (0.5 mM apomorphine)**	84.45	0.75	7.07 ± 0.33	0.9800	445 ± 63***	<0.0001	6
**WT (1 mM apomorphine)**	75.04	0.66	7.12 ± 0.31	0.9405	468 ± 62***	<0.0001	7

Stalk peptide (pG4)-induced G_s activation of ADGRG4

ADGRG4	EC_{50} (nM)	EC_{50} ratio[‡]	pEC50		E_{max}[‖](% of WT)		n[¶]
			mean ± s.e.m.[§]	*P* value	% of WT[§]	*P* value	

**WT**	98.42	1	7.01 ± 0.19	/	100 ± 8	/	10
**WT (0.2 mM apomorphine)**	83.28	0.85	7.08 ± 0.33	0.9955	417 ± 58***	<0.0001	6
**WT (0.5 mM apomorphine)**	90.91	0.92	7.04 ± 0.33	0.9995	347 ± 51**	0.0005	6
**WT (1 mM apomorphine)**	509.46	5.18	6.29 ± 0.31	0.1755	443 ± 57***	<0.0001	6

Stalk peptide (pG6) with 0.2 mM apomorphine-induced G_s activation of ADGRG6

**Mutants†**	**EC_{50} (μM)**	**EC_{50} ratio‡**	**pEC_{50}**		**E_{max}‖ (% of WT)**		**n¶**	**Expression# (% of WT)**	
			**mean ± s.e.m.§**	***P* value**	**% of WT§**	***P* value**		**% of WT§**	***P* value**
**WT**	0.57	1	6.24 ± 0.19	/	100 ± 9	/	7	100	/
**N903$^{2.52}$A**	18.23	32	4.74 ± 0.29*	0.0019	124 ± 18	0.6859	5	84 ± 1***	<0.0001
**W979$^{4.50}$A**	133.00	234	3.88 ± 0.30***	<0.0001	164 ± 35	0.1016	6	64 ± 2***	<0.0001

Stalk peptide (pG6) -induced G_s activation of ADGRG6

**Mutants†**	**EC_{50} (μM)**	**EC_{50} ratio‡**	**pEC_{50}**		**E_{max}‖ (% of WT)**		**n¶**	**Expression# (% of WT)**	
			**mean ± s.e.m.§**	***P* value**	**% of WT§**	***P* value**		**% of WT§**	***P* value**
**WT**	7.14	1	5.15 ± 0.20	/	100 ± 9	/	10	100	/
**N903$^{2.52}$A**	1.15	0.16	5.94 ± 0.28	0.0556	109 ± 13	0.8091	6	91 ± 7	0.2972
**W979$^{4.50}$A**	1.22	0.17	5.91 ± 0.28	0.0832	92 ± 11	0.8479	5	70 ± 5*	0.0025

†All mutations were introduced in the wild-type receptor.

‡The EC_{50} ratio ($EC_{50(mutant)}/EC_{50(WT)}$) represents the shift between the wild-type and mutant curves, and characterizes the effect of the mutations on G_s activation.

§Data are mean ± s.e.m. from at least three independent experiments. **P*<0.05, ***P*<0.001, ****P*<0.0001 by one-way analysis of variance followed by Dunnett's post-test compared to the response of wild type.

‖The maximal response is reported as a percentage of the maximum effect at the wild type.

¶Sample size, the number of independent experiments performed in technical duplicate.

#Protein expression levels of ADGRG6 constructs at the cell surface were determined in parallel by flow cytometry with an anti-Flag antibody (ABclonal) and reported as per cent compared to the wild type from at least three independent measurements.
